# Human fecal microbiota transplantation attenuates high dietary oxalate-induced renal calcium oxalate crystal depositions in rats via repairing *Allobaculum-*related gut barrier damage

**DOI:** 10.1128/msystems.00810-25

**Published:** 2025-08-25

**Authors:** Yuhao Zhou, Min Lei, Weihong Cai, Zhenglin Chang, Lingyue An, Shike Zhang, Daqiang Wei, Renjie Jiao, Jie Gao, Yangzhi Xu, Hui Yang, Mingzhao Zhu, Jiabao Cao, Shujue Li, Xiaolu Duan, Wenqi Wu

**Affiliations:** 1 Department of Urology, The Second Affiliated Hospital of Guangzhou Medical University, Guangzhou, Guangdong, China; 2 Guangdong Key Laboratory of Urology, The First Affiliated Hospital of Guangzhou Medical University, Guangzhou, Guangdong, China; 3 Sino-French Hoffmann Institute, School of Basic Medical Sciences, Guangzhou Medical University 655509, Guangzhou, Guangdong, China; 4 Department of Gastroenterology, The Second Affiliated Hospital of Guangzhou Medical University, Guangzhou, China; 5 Key Laboratory of Epigenetic Regulation and Intervention, Institute of Biophysics, Chinese Academy of Sciences 53015, Beijing, China; 6 CAS Key Laboratory of Pathogenic Microbiology and Immunology, Institute of Microbiology, Chinese Academy of Sciences 85387, Beijing, China; 7 Department of Urology, The First Affiliated Hospital of Guangzhou Medical University, Guangzhou, China; Southern Medical University, Guangzhou, Guandong, China

**Keywords:** calcium oxalate crystal depositions, healthy human-sourced fecal microbiota transplantation (hFMT), gut microbiota, intestinal barrier damage

## Abstract

High dietary oxalate (HDOx) diet is a key factor in kidney stone formation, with gut microbiota playing a significant role. Although several studies have indicated that fecal microbiota transplantation from healthy animals can effectively reduce renal calcium oxalate (CaOx) depositions in rats, the gut microbiota composition between human and animal still remains different. This study aims to explore the effect and underlying mechanisms of healthy human-source fecal microbiota transplantation (hFMT) on CaOx crystal depositions, providing new evidence for its potential clinical application in hyperoxaluria and kidney stone treatment. First, fecal microbiota were screened and collected from healthy individuals and transplanted into rats fed with hydroxyproline. We found that hFMT effectively inhibited crystal depositions and kidney injury induced by HDOx diet, regardless of antibiotic pretreatment. Additionally, 16S rDNA sequencing of gut microbiota identified that hFMT treatment reversed HDOx-induced gut microbiota composition change, particularly restoring the abundance and ecological network of *Allobaculum*, which was a key indicator genus associated with CaOx crystal depositions. Compared to controls, the abundance of *Allobaculum* was increased in fecal samples from kidney stone patients and another rat model fed with potassium oxalate. Mechanistically, hFMT markedly attenuated HDOx-induced intestinal barrier disruption to reverse the formation of CaOx crystallization. These findings suggest that HDOx diets could significantly influence the rat gut microbiota. hFMT effectively reduces HDOx-induced renal CaOx crystal depositions and kidney injury via repairing *Allobaculum*-related gut barrier damage in rats. These findings underscore the potential of hFMT as a therapeutic strategy for hyperoxaluria and kidney stone treatment.

**IMPORTANCE:**

This study investigated that healthy hFMT could serve as a novel strategy to inhibit kidney CaOx deposition induced by HDOx diet. By transplanting healthy human gut microbiota into HDOx rats, we found that hFMT significantly reduced CaOx crystal depositions and kidney damage. The treatment also restored the gut microbiota composition, particularly the abundance of *Allobaculum*, a genus closely associated with CaOx crystal depositions. Importantly, hFMT restored intestinal barrier function, providing a new mechanistic insight into the gut-kidney axis in kidney stone formation. These findings highlight hFMT’s potential as a therapeutic strategy for managing hyperoxaluria and kidney stone, offering a promising alternative to traditional treatment.

## INTRODUCTION

Nephrolithiasis, commonly known as kidney stone, is a worldwide disease with high incidence and recurrence rates, affecting a substantial portion of the global population and posing a persistent challenge for urologists (1, 2). Calcium oxalate (CaOx) stone is the most common type, comprising approximately 80% of all kidney stone (3). Hyperoxaluria is one of the key reasons for kidney CaOx stone formation that is marked by elevated urinary oxalate levels, while high oxalate intake (high dietary oxalate [HDOx]) is an essential factor leading to hyperoxaluria (4, 5). Though recent studies have emphasized the crucial role of gut microbiota in regulating oxalate balance and CaOx stone formation, the exact underlying mechanisms remain poorly understood (6).

The gut microbiome plays a critical role not only in food digestion and nutrient extraction but also in influencing the immune responses, infection prevention, and drug metabolism in the host (7). Current studies have emphasized the roles of the gut microbiome in influencing the development of diseases such as kidney disease, diabetes, and inflammatory bowel disease. Recently, studies have reported that the HDOx diet, including hydroxyproline (Hyp), potassium oxalate (K_2_Ox) diet, or ethylene glycol-induced gut microbiota dysbiosis, contributes to CaOx crystal depositions by disrupting intestinal oxalate, altering metabolite production, and damaging the intestinal barrier (8, 9). However, the specific mechanism of the gut microbiota remains unclear.

Fecal microbiota transplantation (FMT) is a promising treatment to restore the gut microbiota balance by transferring fecal bacteria from healthy donors. This innovative therapy has shown efficacy in a variety of diseases, such as *Clostridium difficile* infection, inflammatory bowel disease, irritable bowel syndrome, and metabolic syndrome (10, 11). Therefore, restoring the diversity and abundance of the gut microbiota through FMT is considered to be an effective method for preventing stone formation (12, 13). Although several studies have demonstrated that FMT from healthy rats or guinea pigs can inhibit CaOx crystal depositions, the effects and underlying mechanisms of FMT from healthy humans remain unknown (9, 14, 15).

In this study, we investigated the gut microbiota change induced by the HDOx diet, as well as the effect and underlying mechanism of FMT from healthy human on renal CaOx crystal depositions and gut microbiota alterations induced by HDOx in rats. The results revealed that healthy human-source fecal microbiota transplantation (hFMT) could effectively reduce HDOx-induced renal CaOx crystal depositions and kidney injury in rats, which were associated with the remedial effect of hFMT on *Allobaculum*-related gut barrier damage, thus providing new evidence for the potential clinical application of hFMT in hyperoxaluria and kidney stone treatment.

## MATERIALS AND METHODS

### Experimental animals

Sprague–Dawley rats (male, 7 weeks old) were obtained from Guangdong Experimental Animal Center (Guangzhou, Guangdong, China). Rats were fed with sterilized water and food freely in a specific pathogen-free room and randomly divided into five groups, with each group comprising 10 rats. One week after acclimatization, rats in different groups were fed with different diets. The weights of rats were recorded every week.

### Experimental design and sample collection

**Fig 1:**
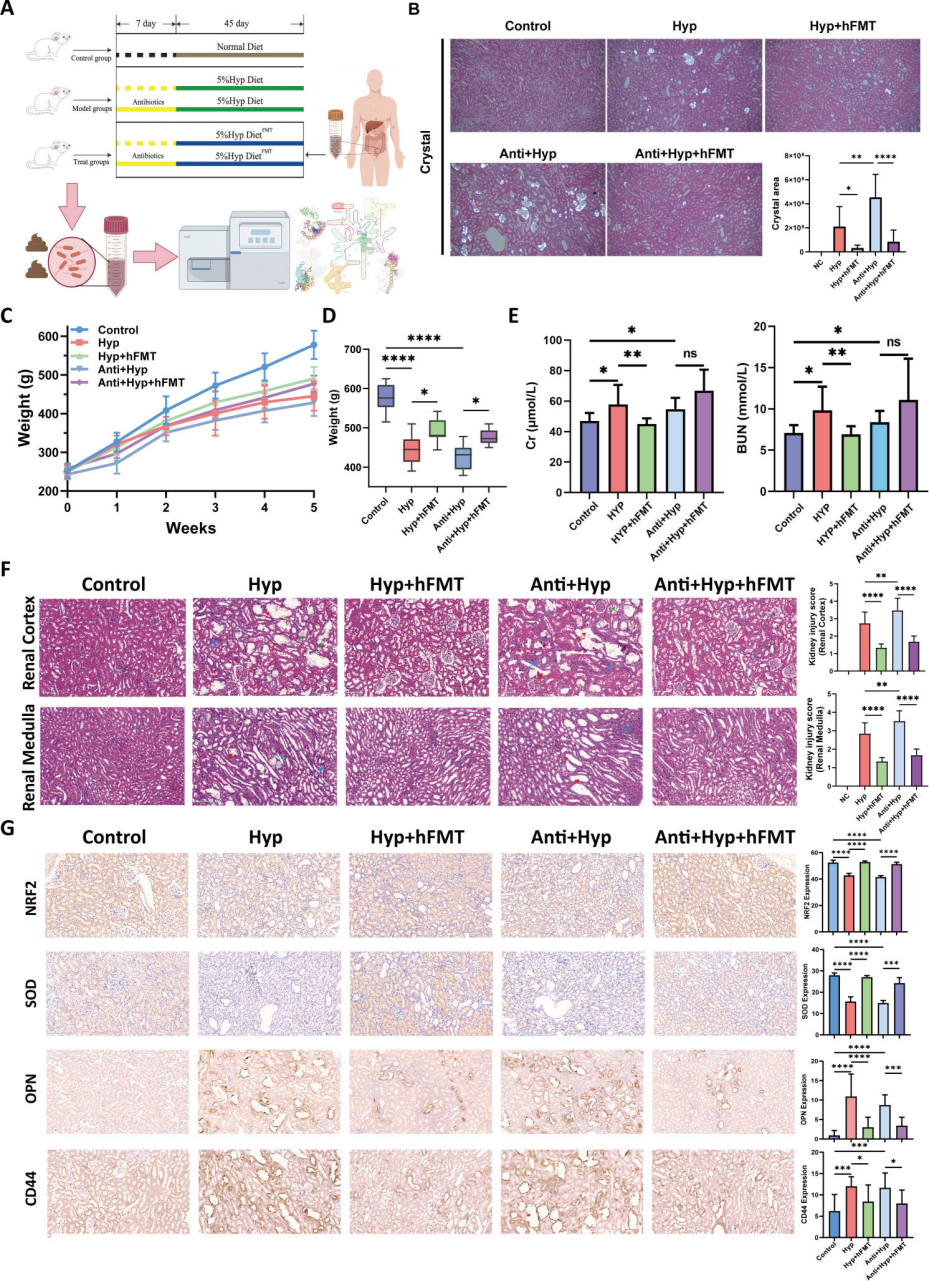
hFMT mitigated HDOx-induced renal CaOx crystal depositions and injury in rats. (**A**) Experimental design of antibodies and hFMT treatment. hFMT, healthy human-source fecal microbiota transplantation; Hyp, hydroxyproline. (**B**) Kidney CaOx crystal depositions in each group were observed by using a polarizing microscope, complemented by statistical graph describing the deposition area. Original magnification: ×400. (**C and D**) The weight changes in each group were assessed at various time points, and the statistical analysis was conducted at the final time point. (**E**) Changes in Scr and BUN levels among different groups. BUN, blood urea nitrogen; Scr, serum creatinine. (**F**) The hematoxylin–eosin (HE) staining results display kidney cortex injury and medulla injury in each group, complemented by statistical graphs describing the HE score. (**G**) Immunohistochemical staining and quantification for indicated proteins in the kidney of rats. Original magnification: ×400. *P < 0.05, **P < 0.01, ***P < 0.001, ****P < 0.0001. ns, no significance.

The experimental design, as depicted in Fig. 1A (see also Fig. 6A), aimed to induce hyperoxaluria and CaOx crystal depositions in rats through HDOx feeding. A rat hyperoxaluria model was established through feeding chow containing 5% Hyp or 5% K_2_Ox according to previous reports (16, 17). The groups were labeled as follows: the control group and normal group (fed with standard diet), the Hyp group (fed with 5% Hyp), the K_2_Ox group (fed with 5% K_2_Ox), and the Anti + Hyp group (gavaged with antibiotic cocktail for a week before feeding with HDOx diet). The antibiotic cocktail consisted of metronidazole (100 mg/kg), vancomycin (50 mg/kg), ampicillin (100 mg/kg), and neomycin sulfate (100 mg/kg).

### Healthy human-source fecal microbiota transplantation (hFMT)

Donor screening and bacterial solution preparation were performed at the Department of Gastroenterology, Second Affiliated Hospital of Guangzhou Medical University. The inclusion and exclusion criteria for healthy human fecal donors were established via three rigorous, sequential screening rounds (18).

Before HDOx diet exposure, fecal samples from healthy individuals were collected, combined, and resuspended in precooled sterile physiological saline (250 mg/mL) and centrifuged at 1,000 rpm for 10 min at 4°C under sterile conditions. The bacterial solution was mixed with 10% sterilized glycerol and stored at −80°C until transplantation. The glycerol in the upper layer was removed by centrifugation during FMT and transplanted into each group of recipient rats by gavage, 200 µL/2 days. Forty-five days after feeding with HDOx, all rats were euthanized by intraperitoneally injecting 2% pentobarbital. Serum was collected by centrifugation (10 min 1,500 × *g*, 4°C); kidneys and intestines were collected for preparing paraffin sections.

### Renal CaOx crystal depositions and injury evaluation

To examine the crystal depositions and histological changes in renal tissues, paraffin-embedded kidney tissues were sectioned into 6 µm slices and stained with hematoxylin–eosin. CaOx crystal depositions in the kidneys were observed by using a polarizing microscope, and the crystal area was quantified by WZCamera software. Five random visual fields were selected for each hematoxylin–eosin-stained kidney section. The percentage of damaged renal tubules was assessed as follows: 0, no damage; 1, <25% damage; 2, 25%–50% damage; 3, 50%–75% damage; and 4, >75% damage (19).

### 16S rDNA sequencing

Microbial DNA was extracted using the HiPure Stool DNA Kits (Magen, Guangzhou, China). The 16S rDNA target region of ribosomal gene was amplified by PCR with the following conditions: 95°C for 5 min, followed by 30 cycles at 95°C for 1 min, 60°C for 1 min, 72°C for 1 min, and a final extension at 72°C for 7 min using the V3–V4 region-specific primers with the following sequences: forward 5′CCTACGGGNGGCWGCAG3′(341F) and reverse 5′GGACTACHVGGGTATCTAAT3′ (806R). The PCR reactions were performed in triplicate, with a 50 µL mixture containing 10 µL of 5× Q5 Reaction Buffer, 10 µL of 5 × Q5 High GC Enhancer, 1.5 µL of 2.5 mM deoxynucleotide triphosphate, 1.5 µL of each primer (10 µM), 0.2 µL of Q5 High-Fidelity DNA Polymerase, and 50 ng of template DNA. The amplicons were extracted from 2% agarose gels, purified using the AxyPrep DNA Gel Extraction Kit (Axygen Biosciences, Union City, CA, USA), and quantified using an ABI StepOnePlus Real-Time PCR System (Life Technologies, Foster City, USA). The purified amplicons were then pooled in equimolar concentrations and subjected to paired-end sequencing (PE250) on an Illumina platform according to standard protocols.

### Microbiota data analysis

The representative operational taxonomic unit (OTU) sequences were assigned to organisms by a naive Bayesian model using the RDP classifier (version 2.2) based on the SILVA database (version 132), the UNITE database (version 8.0), or the ITS2 database (version update 2015), with a confidence threshold of 0.8. The abundance statistics of each taxon were visualized using Krona (version 2.6). The community composition was visualized using a stacked bar plot in the R project ggplot2 package (version 2.2.1). The α-diversity of the gut microbiota was assessed by using the Shannon and Simpson indices, which were calculated in QIIME (version 1.9.1). The β-diversity of each group, which was assessed by using principal coordinate analysis, was generated in the R project Vegan package (version 2.5.3) and plotted in the R project ggplot2 package. Statistical analysis using Welch’s *t*-test, the Kruskal‒Wallis *H* test, Adonis, and the analysis of similarities (ANOSIM) test was performed with the R project Vegan package.

Through the using of LEfSe software, linear discriminant analysis effect size (LEfSe) was applied to identify the differentially abundant taxa with linear discriminant analysis scores exceeding 3.5 within each group. The ecological networks among microbiota with differential abundance were investigated using sparse correlations for compositional data (SparCC). Correlation scores of ≤−0.5 or ≥0.5 with *P* ≤ 0.001 were retained for network inference, and the top 50 pairs are shown in the network.

### Immunohistochemistry and immunofluorescence

The expressions of Osteopontin (OPN), Cluster of Differentiation 44 (CD44), Nuclear factor erythroid-2-related actor 2 (NRF2), and superoxide dismutase (SOD) in the kidney, as well as Zonula occluden-1 (ZO-1) and Occludin in the intestine, were assessed by immunohistochemistry (IHC) staining. Additionally, the expressions of ZO-1 and Occludin were examined using immunofluorescence (IF). IHC and IF procedures were conducted as described previously (20). The following antibodies were employed, including OPN (1:200), CD44 (1:200), ZO-1 (1:200), and Occludin (1:200). Five random visual fields were quantified for each kidney tissue and intestinal section. The quantification of protein expressions was performed using ImageJ software.

### Patient feces collection and quantitative real-time polymerase chain reaction analysis

Feces samples from 30 kidney CaOx stone patients and 30 healthy individuals (without kidney stone) were collected at the Second Affiliated Hospital of Guangzhou Medical University between June 2023 and June 2024 (No. 2023-ks-35). Kidney stone disease was diagnosed by kidney ureter bladder X-ray, urinary system ultrasound, or abdominal computed tomography. The stone composition was confirmed via infrared spectroscopy. Patients with the following conditions were excluded: non-calcium oxalate stone, infectious stone, urine culture positive, urinary system abnormality, renal insufficiency, hyperthyroidism, hyperparathyroidism, hypertension, diabetes, body mass index of >30, or history of gastrointestinal surgery. Participants were also excluded if they had used antibiotics or immunosuppressants or acquired gastrointestinal diseases within 1 month prior to fecal sampling (21).

Microbial DNA was extracted from feces using the HiPure Stool DNA Kits (Magen). Real-time PCR analysis was performed on the lightCycler 480 system (Roche, Basel, Switzerland) using TB green (Cat #RR420A, TaKaRa) as the fluorescent dye. Specific primers for each target gene were utilized, and their sequences are listed as follows: *Allobaculum-*forward AGCAGTAGGGAATTTTCGTCAATGG and *Allobaculum-*reverse CAGACCATTTCCTTTCTGCTTCCTT. The expression levels of the target markers were normalized to the expression of 16S rDNA.

### Statistical analysis

Statistical analyses were performed using GraphPad Prism (version 8.0), and Student’s *t*-test was used to evaluate differences in protein expressions. *P* < 0.05 was considered to indicate a significant difference.

## RESULTS

### hFMT mitigated HDOx-induced renal CaOx crystal depositions and injury in rats

First, we explored the effect of healthy hFMT on HDOx-induced renal CaOx crystal depositions and injury in rats. As shown in Fig. 1B, the rat in the antibiotic pretreatment group (Anti + Hyp group) had more CaOx crystal depositions than the Hyp group. Moreover, hFMT treatment significantly reduced the CaOx crystal area induced by HDOx. On the last day, compared to the control group, rats in the Hyp and Anti + Hyp groups exhibited significantly lower weight, whereas rats in both the Hyp + hFMT and Anti + Hyp + hFMT groups exhibited obvious weight increases (Fig. 1C and D). Moreover, both the Hyp and Anti + Hyp groups displayed elevated serum creatinine (Scr) and blood urea nitrogen (BUN) levels compared to the control group, whereas hFMT treatment significantly attenuated the Hyp-induced increases in Scr and BUN levels (Fig. 1E; Table S1).

As kidney injury caused by hyperoxaluria and/or CaOx crystal is an important factor for kidney stone formation, we then investigated the effect of hFMT on HDOx-induced kidney injury. Compared to the Hyp group, the Anti + Hyp group displayed severe renal tubular lumen dilatation, cast formation, epithelial necrosis, and loss of the brush border, indicating kidney injury in both the cortex and medulla (Fig. 1F). Additionally, as shown in Fig. 1G, the expression levels of SOD and NRF2 were significantly decreased in both the Hyp and Anti + Hyp groups compared to that in the control group, and hFMT treatment could obviously reverse these decreases, indicating that hFMT treatment could attenuate kidney oxidative stress damage induced by HDOx. Furthermore, the expression levels of crystal-adhesion proteins, including OPN and CD44, were higher in both the Hyp and Anti + Hyp groups than that in the control group, and these increases were significantly attenuated by hFMT treatment, indicating that hFMT could effectively inhibit HDOx-induced renal injury in rats.

### hFMT significantly restored the gut microbiota composition changes induced by HDOx

**Fig 2:**
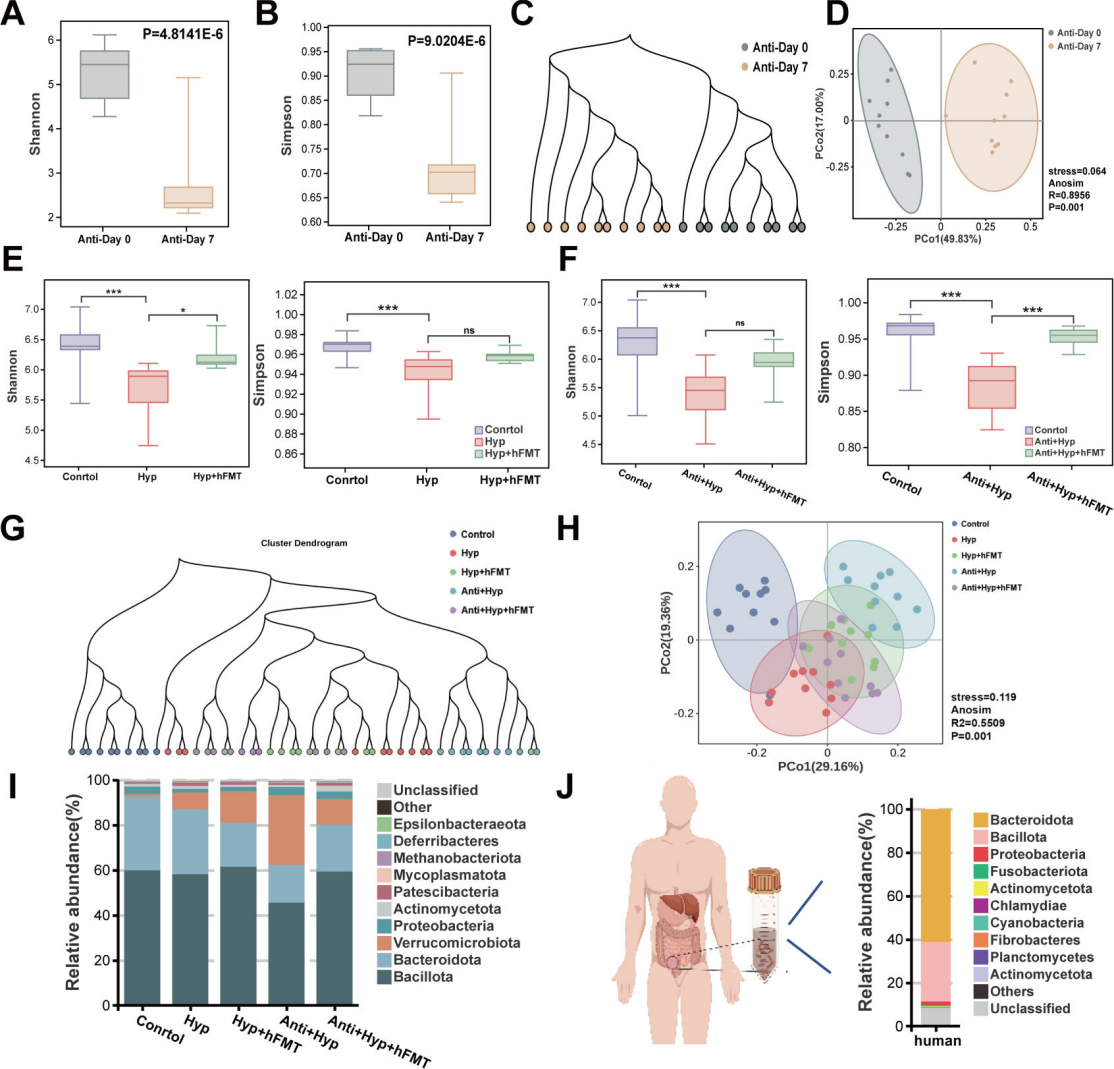
hFMT significantly restored the gut microbiota composition changes induced by the HDOx diet. (**A and B**) The α-diversity of gut microbiota for the Anti-Day 0 and Anti-Day 7 groups, including Shannon and Simpson indices. (**C**) The unweighted pair group method with arithmetic mean (UPGMA) clustering tree indicates the sample relationships between the Anti-Day 0 and Anti-Day 7 groups. (**D**) The β-diversity of the Anti-Day 0 and Anti-Day 7 shown by principal coordinate analysis (PCoA) based on Bray–Curtis distance. (**E and F**) Shannon and Simpson α-diversity indices for each group (**G**) The UPGMA clustering tree indicates the sample relationships between each group. (**H**) The β-diversity of each data group shown by PCoA based on Bray–Curtis distance. (**I**) The bacterial structure of each group at the phylum level. (**J**) The bacterial structure of healthy human feces at the phylum level. *P < 0.05, ***P < 0.001. ns, no significance.

Subsequently, 16S rDNA sequencing was performed to analyze the microbial composition of each sample. Compared to the Anti-Day 0 group, antibiotic treatment significantly reduced the α-diversity and caused an obvious separation in β-diversity, indicating the gut microbiota was depleted by the antibiotic cocktail (Fig. 2A through D). Additionally, the richness and evenness of microbial communities in the HDOx groups (Hyp and Anti + Hyp) were significantly lower than those in the control group. Statistically significant difference in β-diversity was observed between the control group and the HDOx group, whereas hFMT treatment could partially reverse the alterations in both α-diversity and β-diversity (Fig. 2E through H). The reliability of the microbial sequencing data was confirmed by index dilution curves (Fig. S1A and B). Furthermore, the 10 most abundant microbes in rats and healthy individuals are shown in Fig. 2I and J and Fig. S1C and D. Notably, compared to the control group, the abundances of *Bacillota* and *Bacteroidota*, which are typically present in the stool of healthy individuals, were both reduced at the phylum level in the HDOx treatment group, but only *Bacillota* abundance was restored by hFMT treatment. These findings indicated that hFMT could significantly reverse HDOx-induced gut microbiota composition changes.

### Microbiota genera could serve as indicators of HDOx-induced CaOx crystal depositions

**Fig 3:**
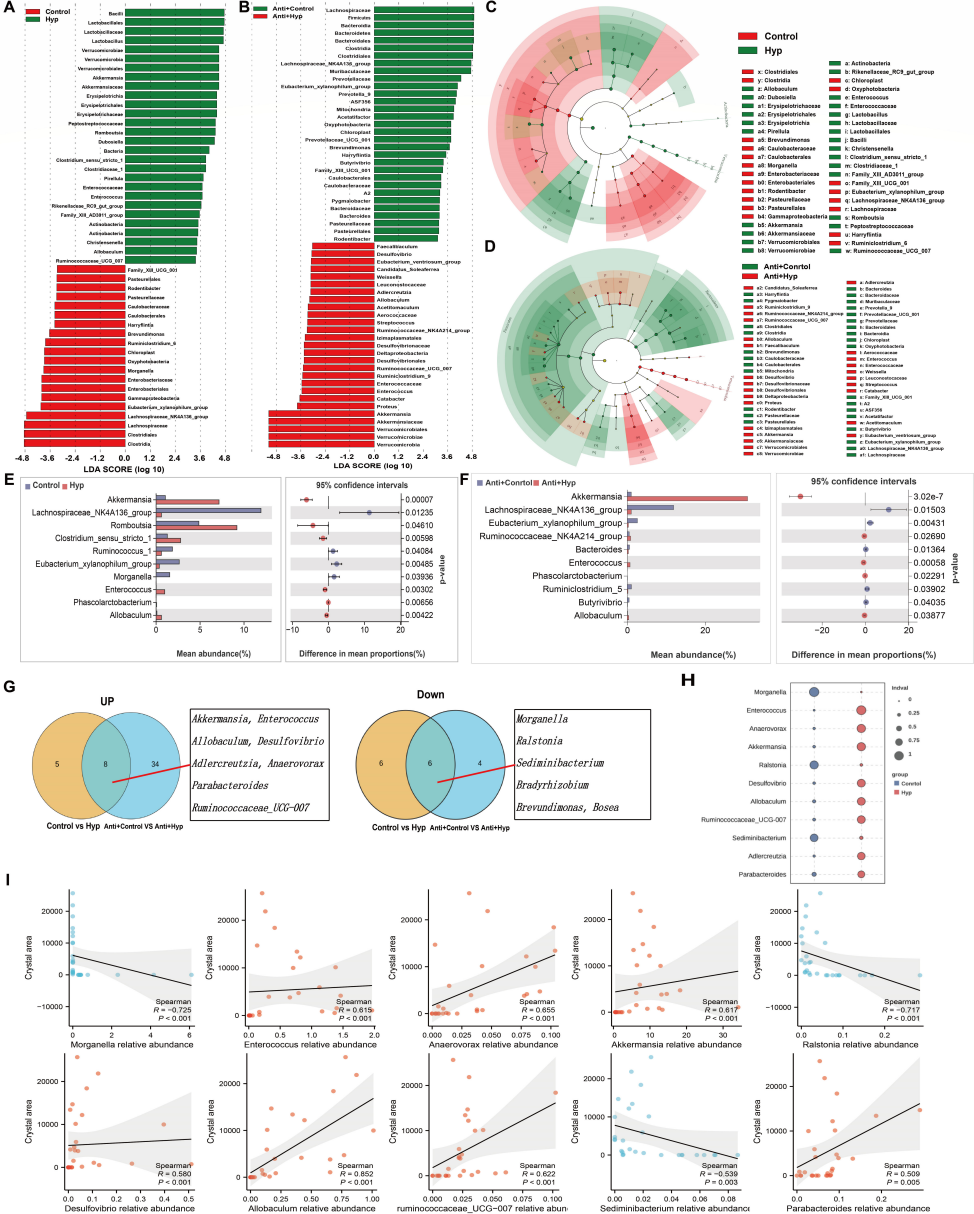
Microbiota genera could serve as indicators of HDOx-induced CaOx crystal depositions. (**A–D**) Linear discriminant analysis effect size analysis diagram and its cladogram show the enriched gut microbiota in each group. (**E and F**) The differential genera between the control group and the HDOx groups were analyzed by Welch’s *t*-test. (**G**) The Venn diagrams indicate the gut bacteria with upregulated or downregulated abundance. (**H**) Indicator value of species in each group at the genus level was analyzed using indicator species analysis. (**I**) The correlation between the genera and the crystal area was analyzed using Spearman’s correlation test.

We then explored the effect of HDOx on the genus composition of gut microbiota. The results of LEfSe revealed that *Akkermansia*, *Enterococcus*, *Allobaculum*, etc., could serve as the key genera in the Hyp group, whereas *Morganella*, *Lachnospiraceae NK4A136* group, etc., were pivotal in the control group. Additionally, *Akkermansia* and *Enterococcus* were found in the Anti + Hyp group, and *Morganella* was prevalent in the Anti + control group. The cladogram distinctly depicted the hierarchical correlations among these discriminative microbes in the groups (Fig. 3A through D). Welch’s *t*-test was then used to identify significant differences in mean abundance between the control and HDOx groups. Specifically, compared to the control group, the relative abundance of *Akkermansia*, *Enterococcus*, *Allobaculum*, *Desulfovibrio*, *Parabacteroides*, *Adlercreutzia*, *Anaerovorax*, and *Ruminococcaceae UCG-007* increased in the HDOx groups, while the relative abundance of *Morganella*, *Ralstonia*, *Sediminibacterium*, *Bradyrhizobium*, *Brevundimonas*, and *Bosea* markedly decreased. None of these changes were affected by antibiotic treatment (Fig. 3E through G). Furthermore, indicator analysis revealed that *Morganella*, *Ralstonia*, and *Sediminibacterium* could serve as the biomarkers for the control group, while *Enterococcus*, *Akkermansia*, *Anaerovorax*, *Desulfovibrio*, *Allobaculum*, *Ruminococcaceae UCG-007*, *Adlercreutzia*, and *Parabacteroides* could be indicator genera for the Hyp group (Fig. 3H). Except for *Adlercreutzia*, the relative abundance of other indicators showed significant correlations with HDOx-induced CaOx crystal deposition area. Notably, the correlation between *Allobaculum* and crystal area was the strongest (*R* = 0.852, *P* < 0.001) (Fig. 3I). These findings suggested that the microbiota genera could serve as indicators of HDOx-induced CaOx crystal depositions in rats.

### hFMT reversed HDOx-induced changes in *Allobaculum* abundance and ecological network

**Fig 4:**
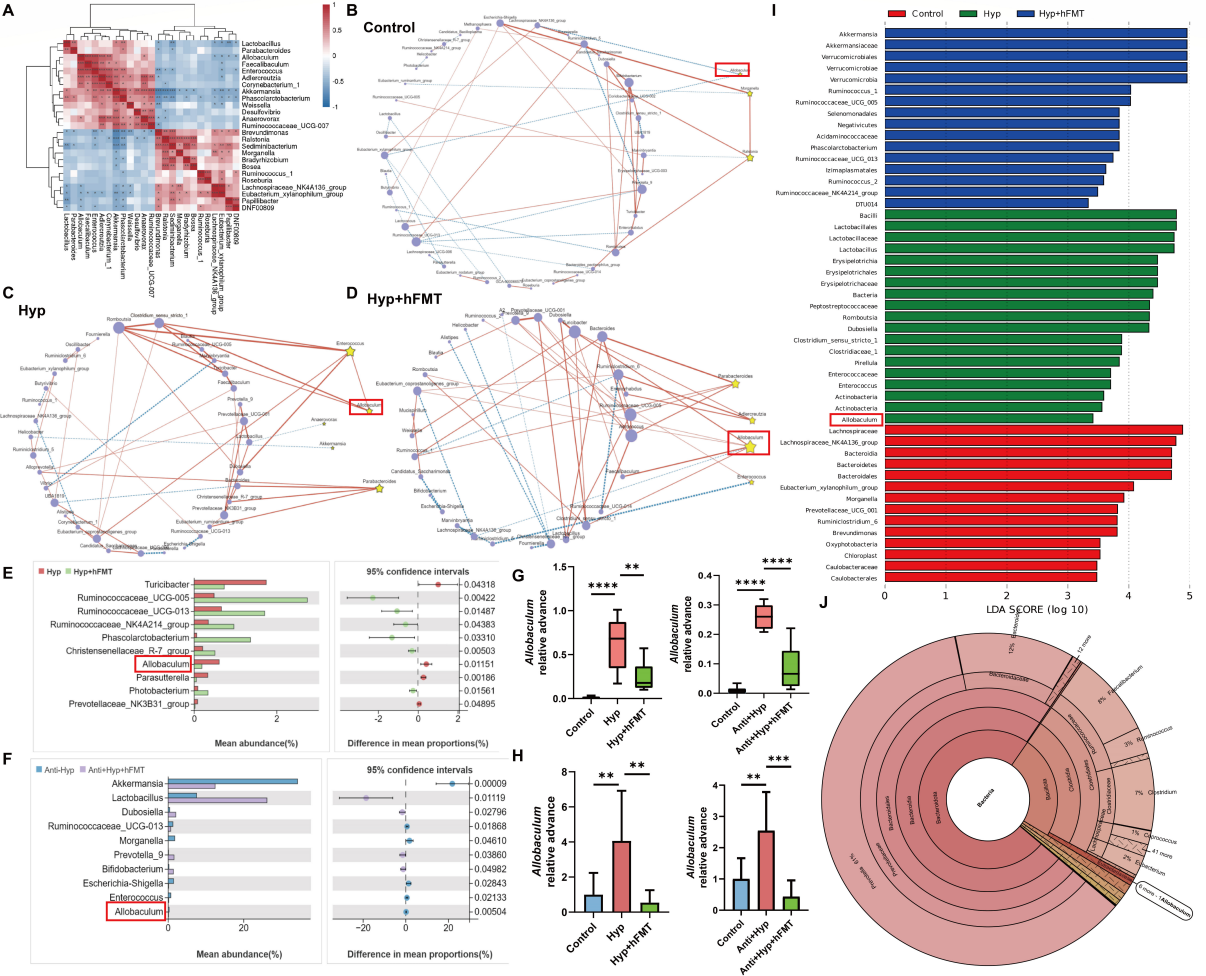
hFMT reversed the HDOx-induced increase in *Allobaculum* abundance and restored its ecological network change. (**A**) The correlation among different gut microbiota was analyzed using Pearson’s correlation test. (**B–D**) The top 50 pairs of the interaction ecological networks among the indicators of each group were measured by SparCC. (**E and F**) The differential genera between the control group and the HDOx groups were analyzed by Welch’s *t*-test. (**G**) The *Allobaculum* relative abundance change in 16S rDNA sequencing. (**H**) The *Allobaculum* relative abundance change via PCR analysis. (**I**) The LEfSe analysis diagram showed the enriched gut microbiota in each group. (**J**) The Krona analysis described the distribution of microbial community structure in the feces of healthy individuals. **P < 0.01, ***P < 0.001, ****P < 0.0001. LDA, linear discriminant analysis.

The interactions among these microbes mentioned above may contribute to disease progression. Correlation analysis revealed that the abundance of *Allobaculum*, a dominant genus, was positively correlated with several microbial genera, including *Enterococcus* (*R* = 0.83, *P* = 6.66E-06) and *Anaerovorax* (*R* = 0.45, *P* = 0.04), whereas it was negatively correlated with the abundance of the *Lachnospiraceae NK4A136* group (*R* = −0.49, *P* = 0.03). Additionally, *Enterococcus* abundance was positively correlated with *Akkermansia* (*R* = 0.61, *P* = 4.42E-03) and negatively correlated with *Sediminibacterium* (*R* = −0.45, *P* = 0.03) (Fig. 4A). Furthermore, we investigated the interaction ecological networks among the indicators of each group based on SparCC. The top 50 interaction pairs, including co-occurrence and co-exclusion interactions, are shown in Fig. 4B through D. In the Hyp group, *Allobaculum* represented a direct positive correlation with *Enterococcus*, and both bacteria exhibited positive correlations with other bacterial genera. In contrast, *Enterococcus* was not significantly correlated with other bacteria in the control group. Moreover, *Allobaculum* was negatively correlated with two probiotic bacteria: *Lachnospiraceae NK4A136* group and *Eubacterium xylanophilum* group (Tables S2 to S4). Additionally, in comparison with those in the Hyp group, the *Allobaculum-*related ecological network in the hFMT group was significantly inhibited, and the interaction between *Allobaculum* and *Enterococcus* was disrupted. These results suggested that *Allobaculum*-mediated ecological networks may play key roles in regulating HDOx-induced CaOx crystal depositions in rats. Furthermore, the increase of *Allobaculum* abundance was reversed by hFMT (Fig. 4E through H). Moreover, *Allobaculum* was identified as the key genus of the Hyp group by LEfSe analysis (Fig. 4I). Additionally, Krona indicated that *Allobaculum* was presented in the feces of healthy individuals (Fig. 4J). Together, these results further indicated that hFMT could reverse the HDOx-induced increase in *Allobaculum* abundance and restored its ecological network change.

### hFMT repaired HDOx-induced gut barrier damage via reversing the increase in *Allobaculum* abundance

**Fig 5:**
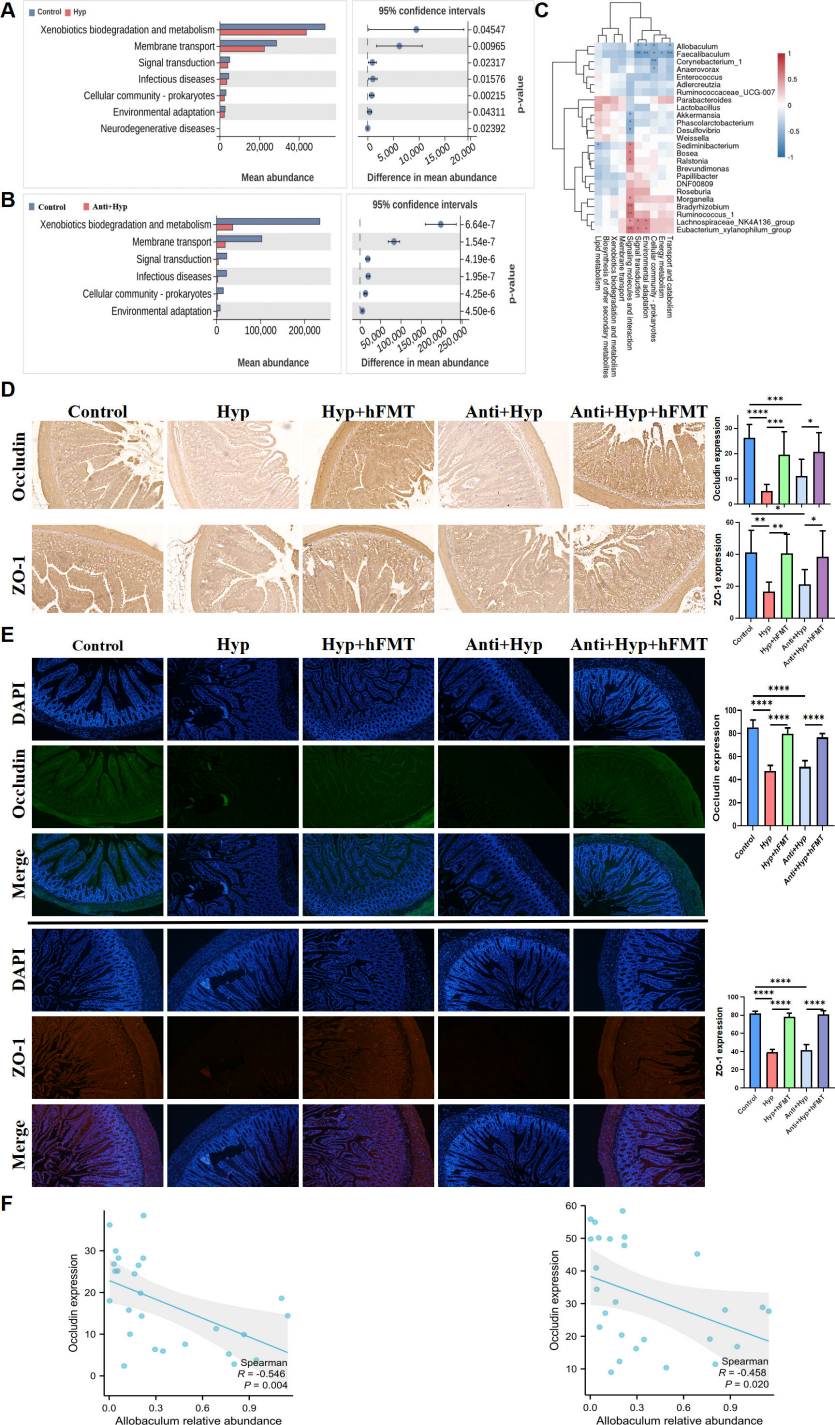
hFMT repaired HDOx-induced gut barrier damage through reversing the increase of *Allobaculum* abundance. (**A and B**) Predict functional changes by using PICRUSt2 analysis. (**C**) The correlation between gut microbiota and functions was analyzed using Pearson’s correlation test. (**D and E**) IHC and IF experiments for the indicated proteins in the intestine of a rat, complemented by statistical graphs describing the positive area. (**F**) Correlation between the indicated protein expression and *Allobaculum* abundance was analyzed using Spearman’s correlation test. Original magnification: ×400. **P < 0.01, ***P < 0.001, ****P < 0.0001. ns, no significance.

Previous studies have shown that ethylene glycol, a precursor to oxalate, can cause intestinal barrier damage and subsequently promote CaOx crystal depositions by influencing gut bacteria (22). Thus, to explore the potential functions of microbial communities implicated in kidney CaOx crystal depositions, PICRUSt2 was utilized to analyze predictive pathway. Compared to the control group, the HDOx diet significantly altered cell signaling pathways, including xenobiotic biodegradation and metabolism, and membrane transport (Fig. 5A and B). The correlations between distinct bacterial genera and specific biological functions are shown in Fig. 5C. Notably, *Allobaculum* was significantly negatively correlated with transport and catabolism. Additionally, compared to the control group, expression levels of ZO-1 and Occludin in gut, the key intestinal tight junction proteins, were obviously decreased in the Hyp groups, and these changes were effectively attenuated by hFMT treatment (Fig. 5D and E). Furthermore, the expressions of ZO-1 (*R* = −0.546 *P* = 0.004) and Occludin (*R* = −0.458 *P* = 0.02) were negatively correlated with *Allobaculum* abundance (Fig. 5F). Together, these results suggested that hFMT could repair HDOx-induced gut barrier damage through reversing the increase in *Allobaculum* abundance.

### *Allobaculum* served as an indicator of K_2_Ox diet-induced CaOx crystal depositions and kidney stone patients

**Fig 6:**
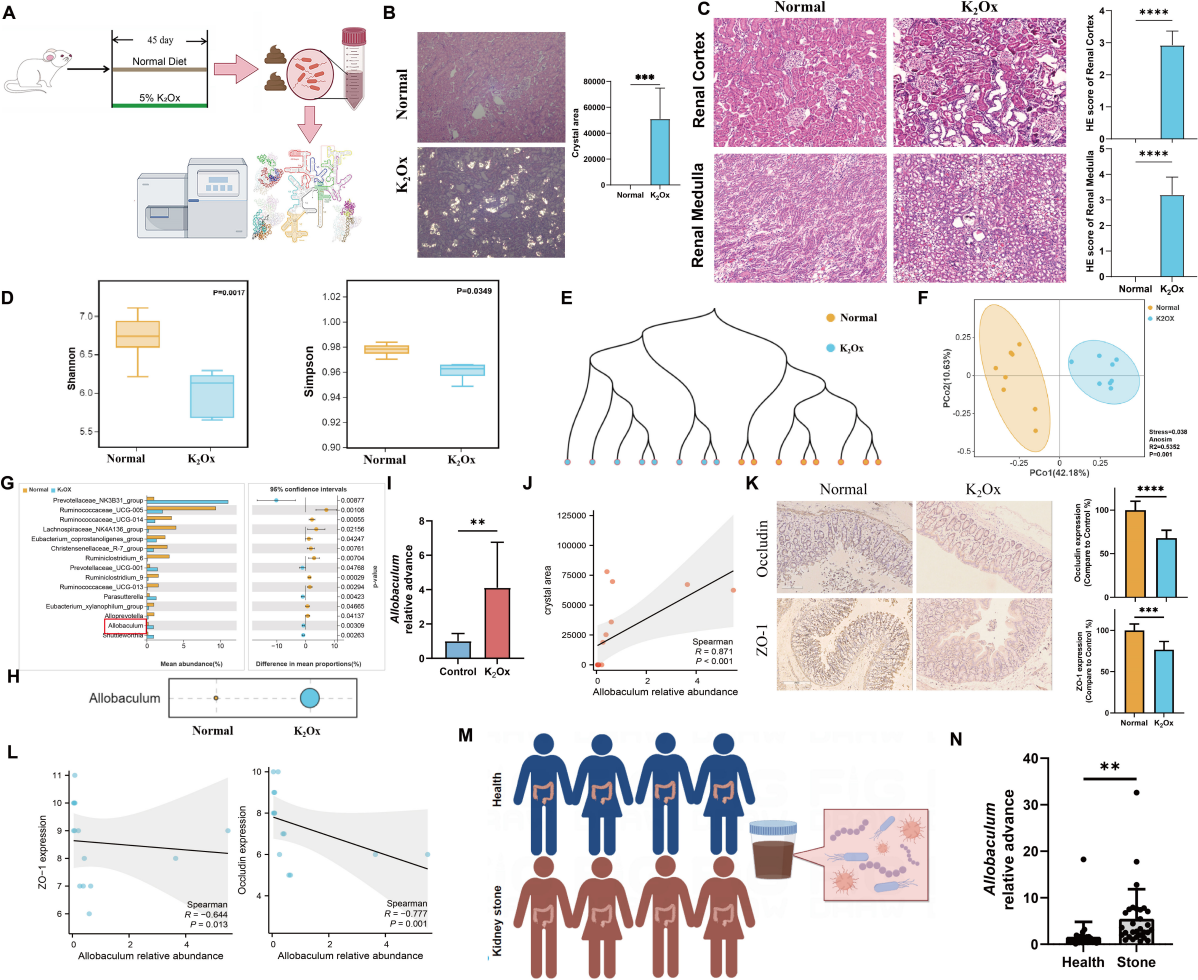
*Allobaculum* served as an indicator of K_2_Ox diet-induced CaOx crystal depositions and kidney stone patients. (**A**) Experimental design of the K_2_Ox diet. (**B**) Kidney CaOx crystal depositions in each group were observed by using a polarizing microscope, complemented by a statistical graph describing the deposition area. Original magnification: ×400. (**C**) The HE staining results display kidney cortex injury and medulla injury in each group, complemented by statistical graphs describing the HE score. (**D–F**) The microbial diversity was measured by α-diversity and β-diversity analyses. (**G**) The differential genera between the control group and the K_2_Ox group were analyzed by Welch’s t test. (**H**) Indicator value of *Allobaculum* in each group at the genus level was analyzed using indicator species analysis. (**I**) The *Allobaculum* relative abundance change via PCR analysis. (**J**) The correlation between *Allobaculum* relative abundance and crystal area was analyzed using Spearman’s correlation test. (**K**) IHC for the indicated proteins in the intestine of the normal and K_2_Ox groups, complemented by statistical graphs describing the positive area. (**L**) Correlation between the indicated protein expression and *Allobaculum* abundance was analyzed using Spearman’s correlation test. (**M**) Feces samples were collected from 30 CaOx stone patients and 30 individuals without kidney stone. (**N**) The *Allobaculum* abundance between the healthy controls and CaOx stone patients. Original magnification: ×400. **P < 0.01, ***P < 0.001, ****P < 0.0001. ns, no significance.

Another hyperoxaluria model induced by feeding 5% K_2_Ox was established to verify the role of *Allobaculum* (Fig. 6A). Compared to the standard diet, the K_2_Ox diet significantly increased CaOx crystal depositions and caused severe kidney injury in rats (Fig. 6B and C). In addition, the results of α-diversity and β-diversity analyses revealed that K_2_Ox feeding significantly reduced the microbial diversity compared to the normal group (Fig. 6D through F; Fig. S1E and F). In comparison with the normal group, *Allobaculum* abundance was increased in the K_2_Ox group. Indicator analysis also identified *Allobaculum* as a biomarker of the K_2_Ox group (Fig. 6G through I). Furthermore, the abundance of *Allobaculum* was positively correlated with the CaOx crystal deposition area induced by K_2_Ox diet (Fig. 6J). These findings further suggested that *Allobaculum* is closely related to the HDOx diet-induced CaOx crystal depositions. Moreover, compared to the normal group, the Occludin and ZO-1 expression levels in the gut significantly decreased in the K_2_Ox group, and their expression levels showed negative correlations with *Allobaculum* abundance (Fig. 6K and L).

Finally, fecal samples from 30 CaOx kidney stone patients and 30 healthy individuals were collected to analyze the relative abundance change in *Allobaculum* (Fig. 6M). As shown in Fig. 6N, the abundance of *Allobaculum* in kidney stone patients was significantly higher than that in healthy individuals. Together, these findings suggested that *Allobaculum* could serve as an indicator of HDOx-induced CaOx crystal depositions.

## DISCUSSION

**Fig 7:**
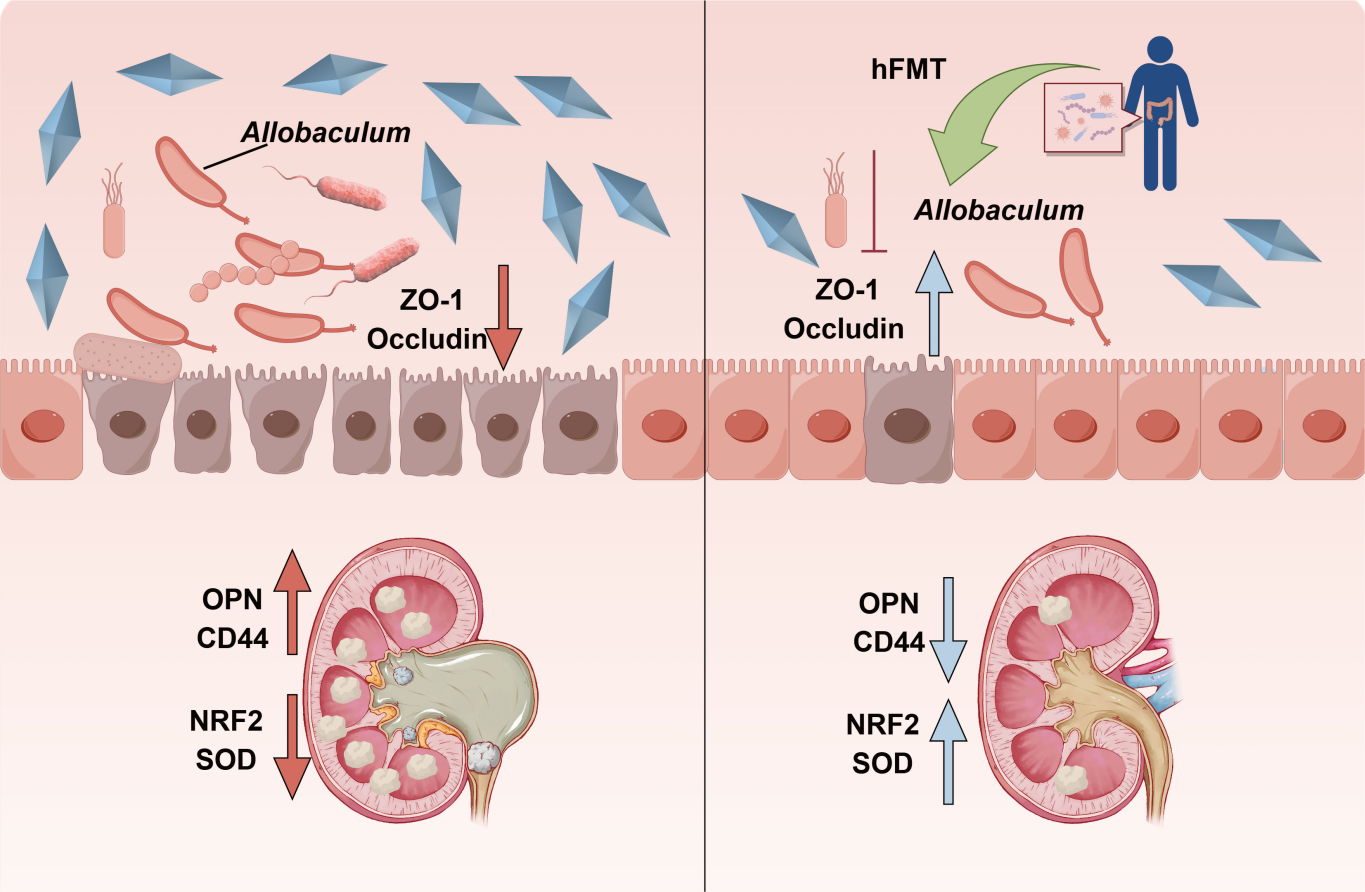
Schematic diagram of hFMT attenuating HDOx-induced CaOx stone formation.

In this study, we investigated the gut microbiota change induced by HDOx diets, as well as the effect and underlying mechanisms of healthy hFMT on HDOx-induced CaOx crystal depositions in rats. The results demonstrated that gut microbial community was significantly influenced by the HDOx diet, and hFMT treatment could effectively reduce HDOx-induced CaOx crystal depositions and kidney injury via repairing *Allobaculum*-related gut barrier damage in rats (Fig. 7).

Dietary oxalate and its precursor intake are well-known to influence urinary oxalate excretion and kidney stone risk. Previous studies indicate that higher oxalate intake increases urinary oxalate levels by 25%–53% (23, 24). While diet is known to shape gut microbiome composition, the effect of microbiota changes induced by HDOx diets on secondary hyperoxaluria remains underexplored. Researchers found that the high nephrolithiasis risk dietary pattern, including HDOx diet, significantly affects CaOx stone formation by regulating gut microbiota homeostasis in patients (25–27). The gut microbiota diversity decreased, and the decreased abundances of the primary phyla *Bacillota* and *Bacteroidota* were observed in HDOx diet group, which were similar to the findings from previous studies on kidney stone patients (21, 24, 28). Furthermore, in the rat supplied with ethylene glycol, a lower diversity of gut microbiota was observed, showing a similar change in gut microbiota diversity as that induced by the Hyp or K_2_Ox diets (29). Through analysis, we found that the indicators of HDOx-induced CaOx crystal depositions, including *Enterococcus*, *Akkermansia*, *Anaerovorax*, *Desulfovibrio*, *Allobaculum*, *Ruminococcaceae UCG-007*, and *Parabacteroides*, had a positive relationship with the crystal area. Among them, the abundance of *Akkermansia* is higher in kidney stone patients compared to healthy individuals. Moreover, the abundance of *Allobaculum* increases in rats with kidney stone induced by an ethylene glycol diet (30–32).

FMT has emerged as a promising therapeutic approach for various diseases, including intestinal inflammation, obesity, and stroke. Animal-sourced FMT has been shown to alter urine chemistry risk factors of kidney stone and prevent CaOx crystal depositions in the kidney by rebalancing the gut microbiota (33, 34). Wang et al. discovered that FMT from healthy guinea pigs effectively decreased CaOx crystal depositions in guinea pig kidneys induced by a 5% potassium oxalate diet via modulating the abundance of *Muribaculaceae* and other oxalate-degrading bacteria (22). However, the effect of healthy human-source fecal microbiota transplantation in inhibiting CaOx crystal depositions in animal models remains unknown. In this study, we found that hFMT also could significantly inhibit CaOx crystal depositions and kidney injury induced by HDOx diet in rats via rebalancing the gut microbiota composition and repairing intestinal damage, suggesting the potential use of hFMT as a therapeutic option for kidney stone treatment.

Although the correlation between oxalate ingestion and gut microbiota alterations has been widely studied, the precise underlying mechanisms remain unclear (8). In the present study, our results showed that HDOx treatment could lead to a decrease in α-diversity and a statistically significant shift in β-diversity of gut microbiota in rats, whereas these changes were obviously restored by hFMT treatment. Importantly, hFMT treatment efficiently reversed the change in *Bacillota*, underscoring the critical role of gut microbiota in regulating CaOx crystal depositions. Furthermore, 10 genera, including *Allobaculum*, *Morganella*, and *Enterococcus*, those showing significant correlations with CaOx crystal depositions, were identified as indicators of kidney CaOx crystal depositions. SparCC-based correlation analysis also revealed that hFMT could reverse the levels of these bacteria, particularly by disrupting the interaction between *Allobaculum* and *Enterococcus*. The crucial role of *Allobaculum* was further confirmed in CaOx stone patients and in another hyperoxaluria model induced by a 5% K_2_Ox diet, highlighting the significant impact of *Allobaculum-*related gut microbiota on CaOx stone formation.

*Allobaculum*, a genus within the phyla *Bacillota*, is thought to be closely associated with the host epithelium in both human and animal intestines. Recent studies indicated that IgA-coated bacteria, including *Allobaculum*, tend to encroach toward the intestinal epithelial cells and can cause intestinal inflammation in colitis mouse models (35–37). Additionally, *Allobaculum* spp. also are implicated in inflammatory processes. For instance, Miyauchi et al. found that *Allobaculum* could adhere to the small intestinal epithelial cells and induce the expansion of inflammatory intestinal T helper 17 cells, which was causally linked to increased susceptibility to experimental autoimmune encephalitis (38). These studies indicated that *Allobaculum* is closely related to the gut barrier damage. In addition to the kidney, the intestinal tract also plays a crucial role in oxalate excretion and absorption. This process relies on paracellular transport facilitated by tight junctions in gastrointestinal epithelial cells. These tight junctions are essential for epithelial barrier function and play vital roles in regulating the transmembrane transport of water, ions, and macromolecules through paracellular pathways, thereby maintaining the dynamic balance of oxalate *in vivo* (29, 39). Previous studies have demonstrated that repairing intestinal barrier damage could effectively reduce kidney CaOx crystal depositions. However, the underlying mechanisms remain unclear (24, 40, 41). Here, we found that *Allobaculum* abundance was positively correlated with HDOx-induced gut barrier damage and kidney CaOx crystal depositions, and hFMT could effectively reverse HDOx-induced *Allobaculum* abundance increase, gut barrier damage, and kidney CaOx crystal depositions, suggesting the crucial role of *Allobaculum* in HDOx-induced gut barrier damage and hFMT-mediated repair.

However, several limitations should be noted. First, while hFMT reversed the HDOx-induced changes in the relative abundance and ecological network of *Allobaculum*, the direct effects on CaOx crystal depositions and gut barrier damage need to be further explored. Additionally, although hFMT effectively reduces kidney CaOx crystal deposition in our study, its clinical translation is challenged by the strict donor screening requirements, undefined patient inclusion criteria and route of administration, and long-term safety (42). Targeted inhibition of *Allobaculum* abundance and function, such as antimicrobial peptides or microbiota modulating agents, could offer a more precise and scalable therapeutic strategy. Together, these efforts will clarify the mechanism of kidney stone formation and pave the way for safer, more reproducible clinical interventions.

In conclusion, our results demonstrated that healthy hFMT could significantly reduce kidney CaOx crystal depositions and injury in rats via repairing HDOx intake-induced gut microbiota disruption. Furthermore, *Allobaculum* could serve as a crucial indicator of HDOx diet-induced CaOx crystal depositions, and hFMT was able to effectively repair *Allobaculum*-related gut barrier damage. Our findings are helpful to provide new experimental evidence for the use of hFMT in the clinical prevention and treatment of CaOx kidney stone. As a novel non-invasive therapy, hFMT holds great potential for the prevention and treatment of kidney stone in the future.

## Data Availability

All data generated or analyzed during this study are included in this published article and its supplementary information files. Data sets not directly provided in the publication are available from the corresponding author upon reasonable request as stated in the Data Availability Statement.

## SUPPLEMENTAL MATERIAL

Fig. S1 msystems.00810-25-s0001.docx Microbial analysis of each group.

Supplemental Tables msystems.00810-25-s0002.docx Tables S1 to S4.

ASM does not own the copyrights to Supplemental Material that may be linked to, or accessed through, an article. The authors have granted ASM a non-exclusive, world-wide license to publish the Supplemental Material files. Please contact the corresponding author directly for reuse.
