# Supplementary material for: Human fecal microbiota transplantation attenuates high dietary oxalate-induced renal calcium oxalate crystal depositions in rats via repairing Allobaculum-related gut barrier damage
Source: mSystems. 2025 Aug 25;10(9):e00810-25. doi: 10.1128/msystems.00810-25 (PMC12455927; doi:10.1128/msystems.00810-25)
Supplement: Fig. S1 — Microbial analysis of each group. [file msystems.00810-25-s0001.docx]

**
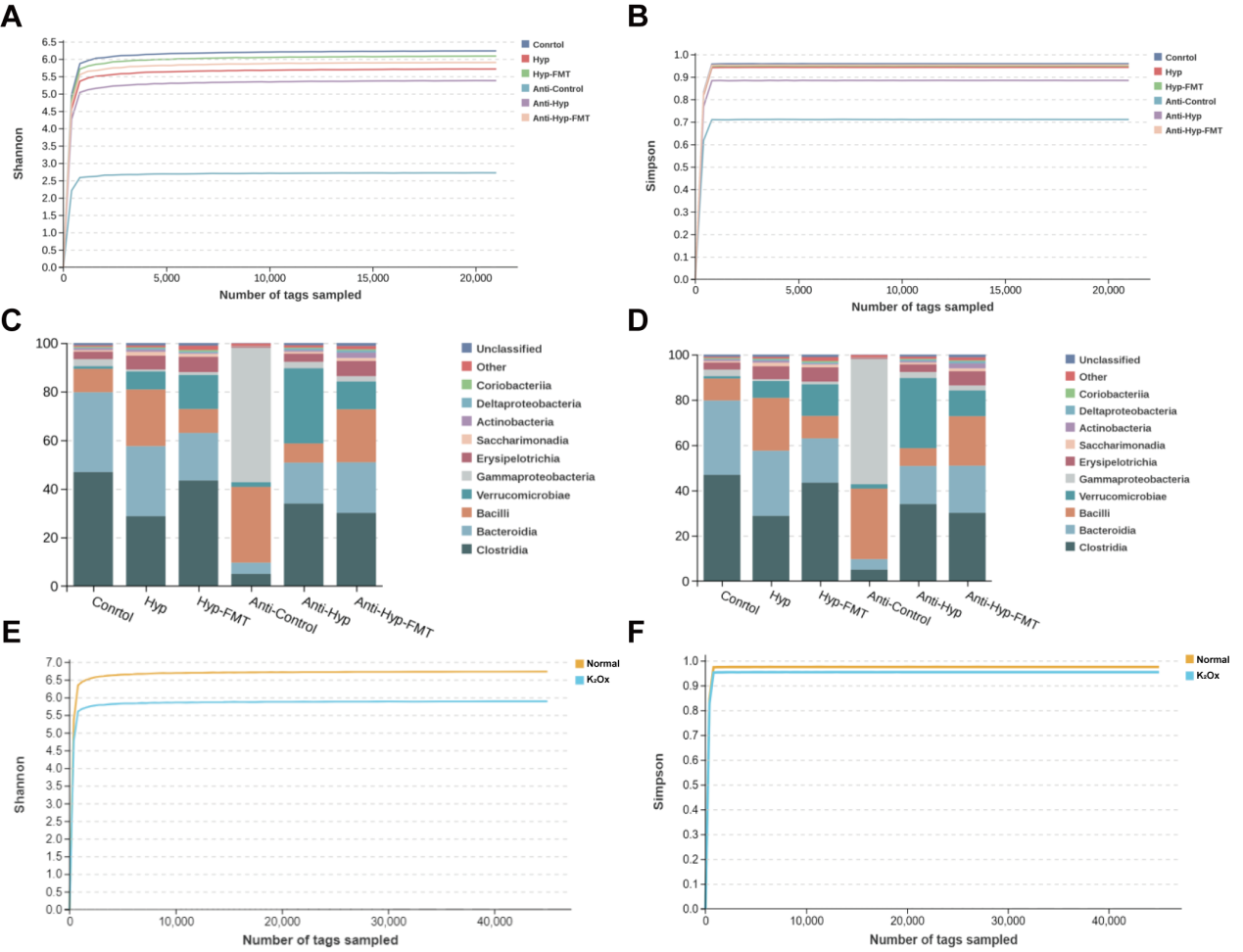
**

**Figure S1. Microbial analysis of each group**

(A, B, E, F). Dilution curves of Shannon and Simpson indexes in each group.

(C, D). The bacterial structure of each group.
