# Supplementary material for: Human fecal microbiota transplantation attenuates high dietary oxalate-induced renal calcium oxalate crystal depositions in rats via repairing Allobaculum-related gut barrier damage
Source: mSystems. 2025 Aug 25;10(9):e00810-25. doi: 10.1128/msystems.00810-25 (PMC12455927; doi:10.1128/msystems.00810-25)
Supplement: Supplemental Tables — Tables S1 to S4. [file msystems.00810-25-s0002.docx]

**Table S1 Biochemical results of serum in each group**

| **Serum index** | **Control** | **Hyp** | **Hyp-FMT** | **Anti-Hyp** | **Anti-Hyp-FMT** |
| --- | --- | --- | --- | --- | --- |
| Cr(umol/L) | 47.07±5.13 | 58.06±17.68 | 44.95±3.79 | 54.69±7.39 | 57.90±12.70 |
| BUN(mmol/mL) | 7.08±0.96 | 9.83+2.86 | 6.92+0.99 | 8.38±1.37 | 11.09+5.00 |
| K(mmol/L) | 4.93±1.88 | 5.44±0.51 | 5.11±0.68 | 5.44±0.74 | 5.29±0.45 |
| Na(mmol/L) | 129.18±43.57 | 144.11±0.99 | 144.40±1.14 | 142.64±2.75 | 143.40±1.44 |
| Cl(mmol/L) | 92.36±30.43 | 100.44±1.39 | 99.95±1.55 | 98.08±1.40 | 98.32±1.56 |
| Ca(mmol/L) | 2.52±0.10 | 2.64±0.11 | 2.59±0.05 | 2.64±0.07 | 2.62±0.06 |
| Phos(mmol/L) | 2.84±0.32 | 2.96±0.39 | 2.46±0.19 | 3.07±0.52 | 2.56±0.20 |
| Mg(mmol/L) | 0.99±0.14 | 0.98±0.15 | 1.02±0.08 | 1.14±0.12 | 1.08±0.17 |

**Table S2. The top 50 pairs of the interaction ecological networks among the indicators of Hyp group were measured by SparCC**

| **var1** | **var2** | **cor** | **p_value** |
| --- | --- | --- | --- |
| *Lactobacillus* | *Prevotellaceae_UCG-001* | 0.873452351 | 0 |
| *Lactobacillus* | *Prevotellaceae_NK3B31_group* | 0.816447741 | 0.04 |
| *Lactobacillus* | *Anaerovorax* | -0.752083462 | 0 |
| *Akkermansia* | *Helicobacter* | -0.75833045 | 0.02 |
| *Romboutsia* | *Dubosiella* | 0.790798615 | 0.04 |
| *Romboutsia* | *Clostridium_sensu_stricto_1* | 0.920835977 | 0 |
| *Romboutsia* | *Eubacterium_coprostanoligenes_group* | 0.751796431 | 0 |
| *Romboutsia* | *Turicibacter* | 0.881184163 | 0 |
| *Romboutsia* | *Enterococcus* | 0.933302262 | 0 |
| *Romboutsia* | *Allobaculum* | 0.824403001 | 0 |
| *Prevotella_9* | *Prevotellaceae_UCG-001* | 0.881418088 | 0 |
| *Prevotella_9* | *Prevotellaceae_NK3B31_group* | 0.766366545 | 0.02 |
| *Dubosiella* | *Enterococcus* | 0.872837374 | 0.02 |
| *Dubosiella* | *Faecalibaculum* | 0.761018013 | 0 |
| *Clostridium_sensu_stricto_1* | *Eubacterium_coprostanoligenes_group* | 0.756414253 | 0 |
| *Clostridium_sensu_stricto_1* | *Turicibacter* | 0.886817763 | 0 |
| *Clostridium_sensu_stricto_1* | *Enterococcus* | 0.861143728 | 0 |
| *Clostridium_sensu_stricto_1* | *Allobaculum* | 0.877594106 | 0 |
| *Eubacterium_coprostanoligenes_group* | *Enterococcus* | 0.810172408 | 0 |
| *Eubacterium_coprostanoligenes_group* | *Corynebacterium_1* | 0.820698077 | 0 |
| *Candidatus_Saccharimonas* | *Alistipes* | 0.808981126 | 0 |
| *Candidatus_Saccharimonas* | *Lachnospiraceae_UCG-006* | 0.830603945 | 0 |
| *Turicibacter* | *Faecalibaculum* | 0.888707346 | 0 |
| *Bacteroides* | *Christensenellaceae_R-7_group* | 0.818853744 | 0 |
| *Bacteroides* | *Parabacteroides* | 0.86228516 | 0 |
| *Bacteroides* | *UBA1819* | -0.767034436 | 0 |
| *Lachnospiraceae_NK4A136_group* | *Eubacterium_xylanophilum_group* | 0.753690613 | 0 |
| *Lachnospiraceae_NK4A136_group* | *Corynebacterium_1* | -0.783773372 | 0 |
| *Prevotellaceae_UCG-001* | *Alloprevotella* | 0.758061457 | 0 |
| *Prevotellaceae_UCG-001* | *Prevotellaceae_NK3B31_group* | 0.837942483 | 0 |
| *Ruminococcaceae_UCG-013* | *Butyrivibrio* | 0.753973038 | 0.02 |
| *Ruminococcaceae_UCG-013* | *Escherichia-Shigella* | -0.8332896 | 0 |
| *Enterococcus* | *Allobaculum* | 0.80611011 | 0 |
| *Ruminococcus_1* | *Butyrivibrio* | -0.775171502 | 0 |
| *Ruminiclostridium_5* | *Eubacterium_xylanophilum_group* | 0.769926538 | 0 |
| *Ruminiclostridium_5* | *Helicobacter* | 0.784847921 | 0.02 |
| *Ruminiclostridium_5* | *UBA1819* | 0.765164873 | 0 |
| *Blautia* | *Ruminococcaceae_UCG-005* | 0.91515414 | 0.02 |
| *Ruminococcaceae_UCG-005* | *Marvinbryantia* | 0.897475633 | 0.02 |
| *Ruminiclostridium_6* | *Oscillibacter* | 0.759188989 | 0 |
| *Ruminiclostridium_6* | *Fournierella* | 0.756872658 | 0 |
| *Parasutterella* | *Lachnospiraceae_UCG-006* | -0.830618311 | 0 |
| *Christensenellaceae_R-7_group* | *Parabacteroides* | 0.846661716 | 0 |
| *Parabacteroides* | *Alloprevotella* | 0.803010893 | 0 |
| *Parabacteroides* | *Eubacterium_ruminantium_group* | 0.835018829 | 0 |
| *Oscillibacter* | *UBA1819* | 0.754492301 | 0.04 |
| *Marvinbryantia* | *Vibrio* | -0.805790596 | 0 |
| *Prevotellaceae_NK3B31_group* | *Eubacterium_ruminantium_group* | 0.759173191 | 0 |
| *Faecalibaculum* | *Vibrio* | 0.763492864 | 0 |
| *Lachnospiraceae_UCG-006* | *UBA1819* | 0.807768362 | 0 |

**Table S3. The top 50 pairs of the interaction ecological networks among the indicators of Control group were measured by SparCC**

| **var1** | **var2** | **cor** | **p_value** |
| --- | --- | --- | --- |
| *Lachnospiraceae_NK4A136_group* | *Eubacterium_xylanophilum_group* | 0.911387245 | 0.000242113 |
| *Lactobacillus* | *Dubosiella* | 0.8640879 | 0.001262878 |
| *Lactobacillus* | *Bifidobacterium* | 0.866870096 | 0.001166692 |
| *Ruminococcaceae_UCG-014* | *Eubacterium_coprostanoligenes_group* | 0.923332589 | 0.000137688 |
| *Romboutsia* | *Turicibacter* | 0.874063356 | 0.00094275 |
| *Eubacterium_coprostanoligenes_group* | *Erysipelotrichaceae_UCG-003* | 0.858744059 | 0.001463602 |
| *Prevotella_9* | *Lactococcus* | 0.91968117 | 0.000165106 |
| *Ruminococcus_1* | *Ruminococcaceae_UCG-005* | 0.86319962 | 0.001294765 |
| *Ruminococcus_1* | *Methanosphaera* | 0.928793111 | 0.000103149 |
| *Morganella* | *Escherichia-Shigella* | 0.986275455 | 1.53E-07 |
| *Turicibacter* | *Clostridium_sensu_stricto_1* | 0.861629754 | 0.001352544 |
| *Ruminiclostridium_6* | *Butyrivibrio* | 0.891578543 | 0.000529401 |
| *Ruminiclostridium_6* | *DNF00809* | 0.932677334 | 8.28E-05 |
| *Dubosiella* | *Bifidobacterium* | 0.999069955 | 3.27E-12 |
| *Dubosiella* | *Prevotellaceae_NK3B31_group* | 0.973064865 | 2.23E-06 |
| *Dubosiella* | *Alloprevotella* | 0.935801462 | 6.87E-05 |
| *Dubosiella* | *Coriobacteriaceae_UCG-002* | 0.998884871 | 6.76E-12 |
| *Clostridium_sensu_stricto_1* | *UBA1819* | -0.895884563 | 0.000452605 |
| *Ruminococcaceae_UCG-005* | *Methanosphaera* | 0.870987718 | 0.001034264 |
| *Ruminococcaceae_UCG-005* | *Coprococcus_2* | 0.983984587 | 2.82E-07 |
| *Bifidobacterium* | *Prevotellaceae_NK3B31_group* | 0.975150378 | 1.62E-06 |
| *Bifidobacterium* | *Alloprevotella* | 0.938666407 | 5.75E-05 |
| *Bifidobacterium* | *Coriobacteriaceae_UCG-002* | 0.998057614 | 6.21E-11 |
| *Marvinbryantia* | *Erysipelotrichaceae_UCG-003* | 0.958622122 | 1.22E-05 |
| *Marvinbryantia* | *Erysipelatoclostridium* | 0.909907782 | 0.000258217 |
| *Bacteroides* | *Helicobacter* | 0.864460923 | 0.001249659 |
| *Butyrivibrio* | *DNF00809* | 0.881693485 | 0.000741284 |
| *Ruminococcus_2* | *Erysipelotrichaceae_UCG-003* | 0.875427869 | 0.000904101 |
| *Ruminococcus_2* | *Erysipelatoclostridium* | 0.920964717 | 0.000155048 |
| *Prevotellaceae_NK3B31_group* | *Alloprevotella* | 0.924308818 | 0.000130965 |
| *Prevotellaceae_NK3B31_group* | *Coriobacteriaceae_UCG-002* | 0.976559657 | 1.28E-06 |
| *Enterorhabdus* | *Bacteroides_pectinophilus_group* | 0.891262073 | 0.000535398 |
| *Enterorhabdus* | *Blautia* | 0.894128782 | 0.000482857 |
| *Enterorhabdus* | *Lachnospiraceae_ND3007_group* | 0.861305412 | 0.001364711 |
| *Enterorhabdus* | *Ruminococcaceae_UCG-008* | 0.867546434 | 0.001144139 |
| *Erysipelotrichaceae_UCG-003* | *Erysipelatoclostridium* | 0.972521206 | 2.41E-06 |
| *Bacteroides_pectinophilus_group* | *Photobacterium* | 0.894939216 | 0.000468715 |
| *Bacteroides_pectinophilus_group* | *Blautia* | 0.977566698 | 1.08E-06 |
| *Bacteroides_pectinophilus_group* | *GCA-900066575* | 0.858696593 | 0.001465482 |
| *Bacteroides_pectinophilus_group* | *Lachnospiraceae_ND3007_group* | 0.959313583 | 1.14E-05 |
| *Bacteroides_pectinophilus_group* | *Ruminococcaceae_UCG-008* | 0.96089242 | 9.76E-06 |
| *Blautia* | *Lachnospiraceae_ND3007_group* | 0.953722576 | 1.90E-05 |
| *Blautia* | *Ruminococcaceae_UCG-008* | 0.955480477 | 1.63E-05 |
| *Alloprevotella* | *Coriobacteriaceae_UCG-002* | 0.934542843 | 7.42E-05 |
| *Lachnospiraceae_UCG-006* | *Anaerotruncus* | 0.937541692 | 6.17E-05 |
| *DNF00809* | *A2* | 0.873608311 | 0.000955903 |
| *GCA-900066575* | *Lachnospiraceae_ND3007_group* | 0.887625534 | 0.000607922 |
| *GCA-900066575* | *Ruminococcaceae_UCG-008* | 0.890138271 | 0.000557093 |
| *A2* | *ZOR0006* | 0.919673296 | 0.000165169 |
| *Lachnospiraceae_ND3007_group* | *Ruminococcaceae_UCG-008* | 0.999805629 | 6.24E-15 |

**Table S4. The top 50 pairs of the interaction ecological networks among the indicators of Hyp+hFMT group were measured by SparCC**

| **var1** | **var2** | **cor** | **p_value** |
| --- | --- | --- | --- |
| *Romboutsia* | *Clostridium_sensu_stricto_1* | 0.836185731 | 0 |
| *Romboutsia* | *Allobaculum* | 0.800278952 | 0 |
| *Lactobacillus* | *Ruminococcaceae_UCG-005* | 0.856237833 | 0 |
| *Lactobacillus* | *Bacteroides* | 0.86578281 | 0 |
| *Lactobacillus* | *Prevotellaceae_UCG-001* | 0.872319404 | 0 |
| *Ruminococcaceae_UCG-014* | *Ruminiclostridium_6* | -0.851120151 | 0 |
| *Ruminococcaceae_UCG-014* | *Aerococcus* | 0.889952062 | 0.02 |
| *Clostridium_sensu_stricto_1* | *Dubosiella* | 0.885178799 | 0 |
| *Clostridium_sensu_stricto_1* | *Turicibacter* | 0.835553947 | 0 |
| *Clostridium_sensu_stricto_1* | *Allobaculum* | 0.83388728 | 0 |
| *Eubacterium_coprostanoligenes_group* | *Lachnospiraceae_NK4A136_group* | -0.827415273 | 0 |
| *Eubacterium_coprostanoligenes_group* | *Ruminiclostridium_6* | 0.799750195 | 0 |
| *Eubacterium_coprostanoligenes_group* | *Allobaculum* | 0.797899616 | 0 |
| *Eubacterium_coprostanoligenes_group* | *Weissella* | 0.839978705 | 0 |
| *Dubosiella* | *Turicibacter* | 0.936215844 | 0 |
| *Lachnospiraceae_NK4A136_group* | *Marvinbryantia* | -0.855415966 | 0 |
| *Lachnospiraceae_NK4A136_group* | *Allobaculum* | -0.797721121 | 0 |
| *Ruminococcaceae_UCG-005* | *Prevotella_9* | 0.807243051 | 0.02 |
| *Ruminococcaceae_UCG-005* | *Bacteroides* | 0.829687153 | 0 |
| *Ruminococcaceae_UCG-005* | *Prevotellaceae_UCG-001* | 0.802243719 | 0.02 |
| *Ruminococcaceae_UCG-005* | *Parabacteroides* | 0.822498067 | 0 |
| *Ruminococcaceae_UCG-005* | *Aerococcus* | 0.900023948 | 0 |
| *Turicibacter* | *Allobaculum* | 0.869616226 | 0 |
| *Turicibacter* | *Adlercreutzia* | 0.837737694 | 0 |
| *Turicibacter* | *Faecalibaculum* | 0.815705081 | 0 |
| *Turicibacter* | *Weissella* | 0.846613163 | 0 |
| *Ruminococcus_1* | *Ruminiclostridium_6* | -0.807576315 | 0 |
| *Ruminococcus_1* | *Bacteroides* | 0.850097995 | 0 |
| *Ruminococcus_1* | *Parabacteroides* | 0.821311136 | 0.02 |
| *Ruminococcus_2* | *Enterococcus* | 0.802306854 | 0.02 |
| *Candidatus_Saccharimonas* | *Escherichia-Shigella* | -0.854240472 | 0 |
| *Candidatus_Saccharimonas* | *Mucispirillum* | 0.84490974 | 0 |
| *Enterococcus* | *Ruminiclostridium_5* | -0.861376562 | 0 |
| *Ruminiclostridium_6* | *Enterorhabdus* | 0.91064971 | 0 |
| *Ruminiclostridium_6* | *Adlercreutzia* | 0.890019566 | 0 |
| *Christensenellaceae_R-7_group* | *Fournierella* | -0.864764719 | 0 |
| *Christensenellaceae_R-7_group* | *Alistipes* | -0.827501895 | 0 |
| *Christensenellaceae_R-7_group* | *A2* | -0.792550924 | 0.04 |
| *Christensenellaceae_R-7_group* | *Helicobacter* | -0.8243389 | 0.02 |
| *Prevotella_9* | *Prevotellaceae_UCG-001* | 0.962680038 | 0 |
| *Prevotella_9* | *Blautia* | 0.827766121 | 0 |
| *Prevotella_9* | *Aerococcus* | 0.832156139 | 0 |
| *Marvinbryantia* | *Ruminiclostridium_5* | -0.831686735 | 0 |
| *Allobaculum* | *Faecalibaculum* | 0.893374394 | 0 |
| *Escherichia-Shigella* | *Bifidobacterium* | -0.855204331 | 0 |
| *Bacteroides* | *Parabacteroides* | 0.91727271 | 0 |
| *Bacteroides* | *Aerococcus* | 0.864677261 | 0 |
| *Prevotellaceae_UCG-001* | *Aerococcus* | 0.832337332 | 0 |
| *Enterorhabdus* | *Adlercreutzia* | 0.863113603 | 0 |
| *Parabacteroides* | *Aerococcus* | 0.85963894 | 0 |
